# Visualisation with treemaps and sunbursts in many-objective optimisation

**DOI:** 10.1007/s10710-018-9329-0

**Published:** 2018-08-07

**Authors:** David J. Walker

**Affiliations:** University of Exeter, Exeter, UK

**Keywords:** Many-objective optimisation, Visualisation, Evolutionary computation

## Abstract

Visualisation is an important aspect of evolutionary computation, enabling practitioners to explore the operation of their algorithms in an intuitive way and providing a better means for displaying their results to problem owners. The presentation of the complex data arising in many-objective evolutionary algorithms remains a challenge, and this work examines the use of *treemaps* and *sunbursts* for visualising such data. We present a novel algorithm for arranging a treemap so that it explicitly displays the dominance relations that characterise many-objective populations, as well as considering approaches for creating trees with which to represent multi- and many-objective solutions. We show that treemaps and sunbursts can be used to display important aspects of evolutionary computation, such as the diversity and convergence of a search population, and demonstrate the approaches on a range of test problems and a real-world problem from the literature.

## Introduction

Visualisation remains an important topic within evolutionary computation and, as many-objective evolutionary algorithms (MaOEAs) continue to mature, the visualisation of solutions to many-objective problems is an important aspect of this [31]. A many-objective optimisation problem comprises four or more competing objectives, such that a solution $\mathbf {x}$ is quantified by an objective vector $\mathbf {y}$ with four or more elements:

1 $$\begin{aligned} \mathbf {y}= (f_1(\mathbf {x}), \ldots , f_M(\mathbf {x})), \end{aligned}$$

where $M\ge 4$. At various stages during the process of solving a many-objective problem with a MaOEA it is desirable to visualise objective vectors. Visualising the objective vectors to such problems is a non-trivial problem because humans are not able to comprehend more than three spatial dimensions. The main motivation behind this work is to facilitate the decision maker’s selection of a final operating solution. In this work the *decision maker* is considered to be the problem owner—the person who wishes to solve the optimisation problem. They are likely from an industrial or scientific background, and do not necessarily have a background in evolutionary computation. Thus, visualisation is a vital part of the optimisation process as it enables the non-expert user to better understand the results they are presented with. In the case of any MaOEA the task of a decision maker is an important one as the result of executing the algorithm is a set of solutions, which are usually incomparable according to measures such as Pareto dominance. Presented with the solution set, the decision maker must select a single solution that can be implemented to solve the problem. The visualisation methods proposed herein are intended to aid the decision maker in this task.

In the past decade, much work has been focussed on the development of methods that can visualise many-objectives. The information that can be extracted from such methods varies depending on the type of visualisation. For example, in some methods the number of dimensions to be visualised is reduced so that a conventional visualisation can be employed (e.g., [7, 46]). Other methods avoid this loss of information by presenting the objective vectors in terms of the full set of objectives (e.g., [11, 12, 15, 20, 25, 36, 36, 46]) or visualising relationships between solutions (for example, conveying which solutions are superior to others) and are constructed in terms of the full objective set (e.g., [48]).

Hierarchies are a convenient structure within evolutionary computation. Examples include the use of trees to represent populations of solutions, such as the *dominated trees* and *non-dominated tree* structures proposed by [19] and the *non-dominated tree* structure proposed in [33]. The example used later in this work builds on the notion of a quad tree [41]. Solutions are often represented as trees in genetic programming, and a tree-based solution representation was used within NSGA-II in [42]. A study [10, 11] used a tree structure to represent the objectives comprising a many-objective problem in order to reduce the dimensionality of the problem. In this paper we explore the use of *treemaps* [26] and *sunbursts* [40]. A pilot study [47] illustrated the potential of using treemaps to visualise many-objective populations, however it identified two problems. First, the treemaps presented therein were based on trees constructed in terms of dominance. It is well known [16] that the dominance relation is poorly suited to comparing many-objective solutions since, assuming an uniformly distributed objective space, the solutions are likely to be mutually non-dominating and thus incomparable. The result of constructing a treemap or sunburst with such a tree is that there is little structure to present in the visualisation and the user does not obtain any significant insight. The second problem involved the layout algorithm selected for the visualisations. The treemaps presented in [47] used a standard square layout, and the dominance relations that were present in the tree (for multi-objective examples) were difficult to observe. In this work, the initial study is extended to make the following novel contributions:

- A new treemap layout algorithm is presented, specifically designed to visualise many-objective populations with dominated solutions, and compared to an existing approach proposed by [26].
- A quad tree from the literature [41] is used as the basis for a many-objective visualisation.
- The well-known sunburst visualisation [40] is used to visualise many-objective populations; demonstrations show that they can be used to convey information about the optimisation characteristics (e.g., convergence and diversity) as well as the solution quality of a mutually non-dominating set.

Therefore, the principal contribution of this paper lies in the application of tree-based visualisations to many-objective populations using datastructures already used within MaOEAs. This work is the first to have considered the visualisation of many-objective populations using treemaps and sunbursts.

Throughout the paper we present results for a selection of optimisation problems, including well known benchmark problems from the DTLZ problem suite [14], benchmark approximation sets proposed in [43] and solutions to a real-world test problem [24]. The remainder of this paper is organised as follows: Sect. 2 presents some relevant background material, describing existing approaches to many-objective visualisation as well as introducing treemaps. Section 4 presents many-objective sunbursts used for visualising mutually non-dominating sets. Section 5 presents a short user experiment of the methods, and Sect. 6 provides an analysis of the properties of the introduced visualisation methods before concluding remarks are made in Sect. 7.

## Background

This section introduces a range of relevant background material, first describing many-objective visualisation in more detail, before introducing treemaps. The methods described throughout this paper are for visualising the solutions generated by MaOEA’s. The task of a MaOEA is to optimise a problem comprising a set of *M* conflicting objectives to which there can be no solution that simultaneously optimises all *M* objectives. Solutions are compared using the dominance relation, whereby solution *i* dominates solution *j* if it is no worse than *j* on any objective and better on at least one. More formally, assuming a minimisation problem without loss of generality:

2 $$\begin{aligned} \mathbf {y}_i \prec \mathbf {y}_j \Longleftrightarrow \forall m (y_{im} \le y_{jm}) \wedge \exists m (y_{im} < y_{jm}). \end{aligned}$$

If neither *i* dominates *j* or *j* dominates *i* then they are said to be *mutually non-dominating*. A solution with no dominating solutions is called *non-dominated*. The goal of a MaOEA is to identify the *Pareto set*, the set of feasible solutions that cannot be dominated. Its objective space image is called the *Pareto front*.

### Many-objective visualisation

As has been outlined, techniques for visualising many-objective populations fall into three categories, all of which contain methods that are useful for visualising such data while suffering from limitations. In the first, the dimensionality of the data is reduced so that conventional visualisation tools can be applied, while in the second novel methods that are capable of visualising the full data are used. The last, the pairwise relationships are highlighted so that preferred solutions can be identified. This paper is concerned with the latter, and we do not discuss visualisations from the first two classes further.

**Fig. 1:**
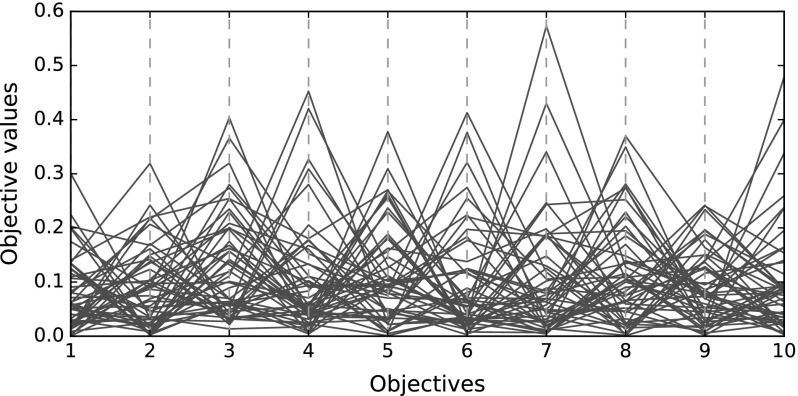
An example parallel coordinate plot. The 50 solutions are mostly indistinguishable from each other

A challenge with visualising data in terms of the full set of objectives is that the visualisations are often too cluttered to observe useful information. Two examples of this are parallel coordinate plots [15, 20, 25] and pairwise coordinate plots [12]. Parallel coordinate plots represent a solution as a line, with the ordinal axis representing the problem objectives, and the abscissa conveying the objective value; an example is shown in Fig. 1. While this is scalable to any number of objectives and solutions the result is often too cluttered to allow a decision maker to extract useful information from it. Pairwise coordinate plots present a population of solutions according to each pair of objectives. This too is scalable, but relationships involving more than two objectives cannot be represented. Heatmaps are also a scalable approach to visualising all of the objectives within a population, and they can be enhanced to better convey the information within the data; for example, the rows and columns (representing solutions and objectives, respectively) can be rearranged to highlight the trade-off between objectives [36, 46]. That said, one of the useful features of a many-objective visualisation is the ability to observe dominance relations between pairs of individuals. That is not easily done using a heatmap, and such information is typically even more difficult to see using feature extraction techniques that have been used to visualise many objective solutions (e.g., self-organising maps [28, 35], generative topographic mappings [6, 18] and neuroscale [18, 32]). Presenting dominance relationships is one of the key aspects of this work.

As a close relative of the tree, methods presenting populations in terms of a graph are relevant to this work. One example of such a method is the Pareto shell visualisation demonstrated in [48]. Therein, a dominance graph is inferred on the population by ranking the individuals with Pareto sorting. Edges are then placed such that if a solution dominates an individual in the immediately inferior shell then $W_{ij}$ indicates the *probability of dominance*, the number of objectives *m* for which $y_{im} < y_{jm}$. That visualisation was enhanced with the use of colour, which is used to convey additional rank information. Various ranking schemes are discussed, and it is shown that the method can reveal useful structural information about the population, for example highlighting poor solutions that are extremely good on one objective and thus difficult to dominate. An extension of this method projected such a graph into the plane for visualisation as a 2-dimensional scatter plot [17].

### Treemaps and sunbursts

A *treemap* is a 2-dimensional visualisation of hierarchical data constructed using a space filling algorithm. They are particularly effective for displaying clusters within data, and have been used in a variety of applications, such as visualising stock market information for identifying fraudulent transactions [23], visualising gene expression data [5], and representing file system hierarchies graphically [44]. Though we are aware of no cases in which treemaps have been used to visualise data arising from evolutionary computation (aside from [47]), they have been used to visualise the evolution of biological organisms [2] and biodiversity [22]. Hierarchies are common within evolutionary computation, examples being solutions to genetic programming problems and hierarchies of solutions defined in terms of the solutions’ relative quality, and for these reasons they are a natural choice for visualisation within evolutionary computation. We note that in their conventional square form treemaps are visually similar to *mosaic* plots [27]. These are not designed to convey hierarchical information, so are not considered herein.

The underlying task in visualising data with a treemap is to convey a sense of the scale or importance of a node by dividing the space within the treemap so that those nodes with high importance are represented by large regions of space, and those smaller or less important nodes receive less space. Various algorithms have been proposed to partition a space in the construction of treemaps. One of the most frequently used is the *squarify* algorithm, which divides a rectangular space into elements as closely as possible have an aspect ratio of 1 [8]. That work suggests that using square elements makes the comparison of pairwise elements’ size simpler, as well as providing a more efficient use of space. Treemaps do not have to be square or rectangular [45]; an alternative algorithm used in [3, 34] uses a Voronoi tessellation to divide the space. This is done by placing seed points that control the placement of irregular regions within the treemap. The regions are arranged so that they correspond to the data being visualised. It is argued by [22] that this type of treemap is more intuitive to a user, in that it is not constrained to fill a rectangular shape and can take more memorable and representative geometries. In this work, our main goal is to represent a multi- or many-objective population with a treemap so that dominance relationships are directly visible in the visualisation. We present two approaches for conveying this information, which are discussed in Sect. 3. An alternative to using the treemaps proposed herein is to exploit the *sunburst* visualisation [40], which divides space according to some quantified values in the way a treemap does, but nodes emanate from the centre of the visualisation. For a comprehensive review of hierarchical visualisation methods see [38].

In addition to the partitioning of the space, additional degrees of freedom can be employed to convey further information about the hierarchy. The obvious candidate is the colour of the node; in their use as a method for visualising clusters of data, treemaps are often coloured according to the cluster membership of a node [9]. The treemap can be further enhanced by using alternative rendering techniques to clarify aspects of the visualisation. An example is the use of “cushioned” nodes [44], which are intended to better highlight the hierarchical aspects of the data being represented. In this work we make use of node colour to represent additional aspects, such as solution quality, along with population convergence and diversity measures.

## Multi-objective populations: treemaps

Before demonstrating how a space-dividing visualisations might be used to convey multi- and many-objective populations we first consider the intended workflow in which they will be incorporated. Two use cases are envisaged—one in which an evolutionary computation practitioner wishes to inspect the solutions within their algorithm’s search population, and the other in which the decision maker selects a solution generated by a MaOEA for implementation. The first use case is an important consideration, as visualising EA operation can enable better selection of algorithm parameters; the state of the search population is an important aspect of the algorithm’s operation. Additionally, interactive EAs are becoming more prevalent, and simple light-weight visualisations of the search population are an important inclusion into the user interface of such algorithms. This section considers the case of search populations, which can (and do) contain dominated solutions, and the following section describes the second use case—those examples deal exclusively with mutually non-dominating solutions.

In order to visualise a population of solutions with a treemap, the population must first be represented as a tree, and we present a method for doing this. The first step is to define a new individual, which we call $\mathbf {n}^r$, that will be the root node in the tree. We constrain that this node must dominate the entire population, so we use the *global best* point in the population to define it. This is the *M*-dimensional vector comprising the minimising objective value on each objective [21]:

3 $$\begin{aligned} \mathbf {n}^r = \left( \min _i y_{i1}, \ldots , \min _i y_{im} \right) . \end{aligned}$$

**Fig. 2:**
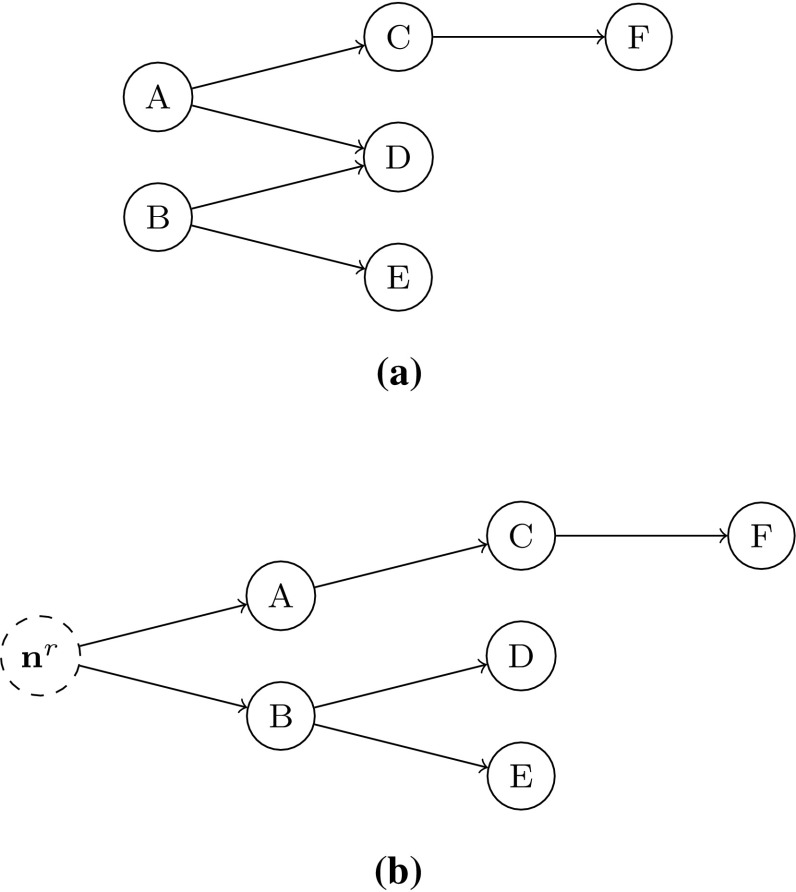
The construction of a tree using dominance. In the top panel, the individuals have been arranged into a graph using Pareto sorting. Pareto sorting is used to induce an ordering over the individuals, edges are placed between those individuals where one dominates another in the immediately inferior Pareto shell. The dominance distance is used to prune edges. All of the parent–child relationships are removed, with the exception of that between which the pairwise dominance distance is closest. A root node $\mathbf {n}^r$ is inserted to represent the ideal point. Thus, the network is reduced to $N-1$ edges

Having defined the tree’s root node, we order the population with Pareto sorting [39]. Pareto sorting begins by identifying the non-dominated solutions, which become the first shell. They are then temporarily discarded, leaving a new non-dominated set. This becomes the second shell, and these solutions are also discarded. Over time, the entire population is assigned to a shell. In Fig. 2, the first shell is comprised of *A* and *B*; the second shell has three members (*C*, *D* and *E*); and the final shell contains individual *F*. This produces a partial ordering of individuals, and as was done in [48] we infer a graph on the population by placing edges between the dominating and dominated individuals in adjacent shells. Additional edges are placed to connect each of the non-dominated individuals to the root. The resulting network is not yet a tree; as shown in Fig. 2, it is possible for an individual to be dominated by two individuals in the superior shell. Using the nomenclature of trees, this means that a node can have two parents. In order to convert the network into a tree, we use the dominance distance [46] to identify which edges should be pruned. The dominance distance is a proper metric, and computes a distance in terms of the number of dominance relations two individuals share with the rest of the population. If the two individuals share most dominance relations then the individuals are said to be close; if they differ on a majority of the relations then they are distant. In order to prune an individual’s excess parents, we compute the dominance distance between the individual and all candidate parents in the superior shell, and retain the edge between it and the parent with which it is closest. The resulting tree structure contains, according to the dominance relation, the “best” solutions (those that are mutually non-dominating) at the highest levels, and the “worst” solutions are the leaf nodes. In terms of the task of a decision maker, this is the most important structural characteristic of the tree as the solutions they are most likely to prefer are those that are mutually non-dominating. The solutions at the lowest levels of the tree are unlikely to be of interest for a decision maker selecting an operating solution. We note that all nodes in the trees in this paper are unweighted.

As discussed above, other algorithms for expressing the dominance relationships between individuals with a tree have been proposed, and we note that the treemap visualisation we now demonstrate is not dependent on the algorithm used to create the tree itself. An approach to visualising a multi-objective population with a treemap was proposed in [47]. Given a tree *T* of multi-objective individuals, we construct a treemap that represents *T* by partitioning a space according to the importance of each node within the tree. Importance is defined in terms of the number of child nodes that the current node has (hence, the number of individuals that the node’s corresponding individual dominates) [47]. In addition to the size of the node, information is conveyed to the decision maker by colouring the nodes according to some relevant scale (the original study demonstrated the use of average rank [4], to provide an additional measure of solution quality, and crowding distance, to provide an indication of population diversity—both are used later in this work).

**Fig. 3:**
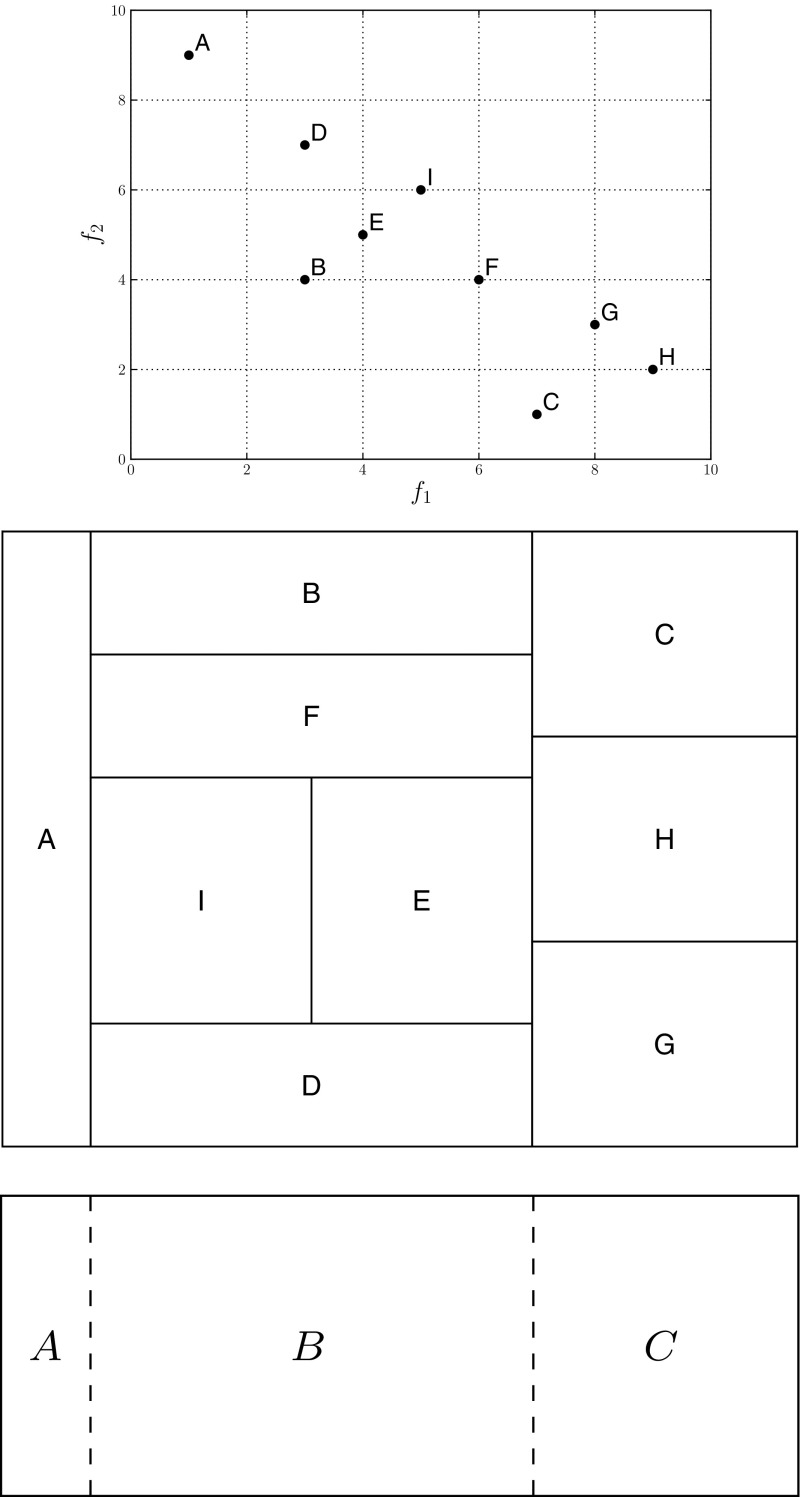
The partitioning of a test population into a treemap. Having constructed a tree, the root node has children *A*, *B* and *C* (the population’s mutually non-dominating individuals). *A* has no children, *B* has children *D*, *E* and *F*, and grandchild *I*, while *C* has children *G* and *H*

Figure 3 illustrates a treemap which visualises an example population of 2-objective individuals. Following the scheme outlined above, a tree is defined over the individuals; non-dominated individuals are child nodes of the artificial root node. A region $r_c$ is defined to specify the extent of the treemap occupied by the current node. The procedure by which the space is partitioned is based on the well-known *slice and dice* method [26]. At the beginning of the partitioning procedure, the current node is the root and $r_c=(0,1,0,1)$, defining the *x* origin, *y* origin, width and height. We initialise the partitioning direction to be vertical, however this is an arbitrary choice.

The first step of partitioning the space is to insert the node corresponding to solution *A*. *A* is placed on the left-hand side of the treemap, and since it dominates no solutions spans the entire vertical extent of $r_c$. Given that there are no further children to add for *A* the next step is to add node *B* to the visualisation. This node has descendants, and $r_c$ is redefined to form the region that is to be occupied by *B* and its descendants. Once *B* itself has been added, its descendants are added recursively. At each layer of recursion (each layer corresponding to a deeper layer of the tree) the partitioning direction is reversed to enhance the clarity of the visualisation. This procedure continues until all of the nodes in *T* have been added to the treemap. We note that in addition to defining the size of a rectangle representing an individual in terms of its number of dominated individuals, we also scale the node according to the Pareto shell to which an individual belongs. This has the effect of better highlighting the strongest individuals, which, from the point of view of a decision maker selecting a final operating point or an individual to use as a parent in the next generation of an interactive optimisation procedure are the individuals likely to be of interest.

**Fig. 4:**
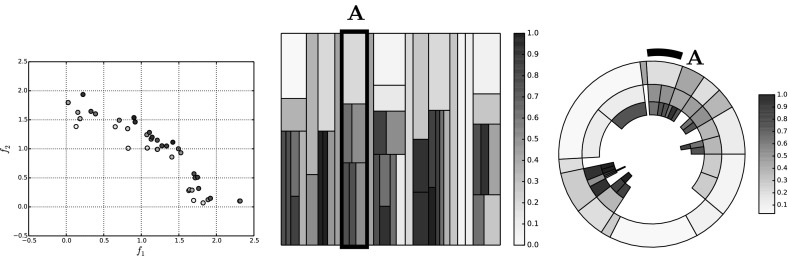
DTLZ2 sample solutions. The left-hand panel shows the objective vectors corresponding to the treemaps in the centre and right panels. Solutions are coloured according to average rank. Region *A* indicates corresponding regions in the treemaps (Color figure online)

Figure 4 illustrates a population of sample solutions to a 2-objective instance of DTLZ2 [14] along with a corresponding treemap of the population in the centre panel (the bottom panel can be ignored for now). The population comprises 50 individuals generated from the feasible space ($x_p \in (0,1), \forall p \in P$, where each solution has *P* parameters). In both visualisations, the individuals have been coloured according to *average rank* [4]; the population is ranked according to each objective, to produce *M* rankings of the population such that $r_{im}$ indicates the rank of the *i*-th individual on the *m*-th objective. From these, the average rank for individual *i* is computed as follows:

4 $$\begin{aligned} r_i = \frac{1}{M}\sum ^M_{m=1} r_{im}. \end{aligned}$$

A low average rank is shown by a light colour, and indicates a good solution. Poor solutions are represented by high average ranks in red. Average rank values have been normalised to the range (0, 1). There has been no effort to place the nodes within the treemap in a particular order; this is discussed shortly. Good solutions are easily observed, from their lighter colour and generally larger node size. We note that there are several non-dominated individuals that do not dominate any other members of the population. From inspecting the scatter plot in the left-hand panel it can be seen that such solutions do not exist. This is an artefact of the edge pruning procedure; these individuals were one of a set of candidate parent nodes for the solutions they dominate, and have a greater dominance distance from the individual than the individual which retained the relationship and became the dominated individual’s parent.

**Fig. 5:**
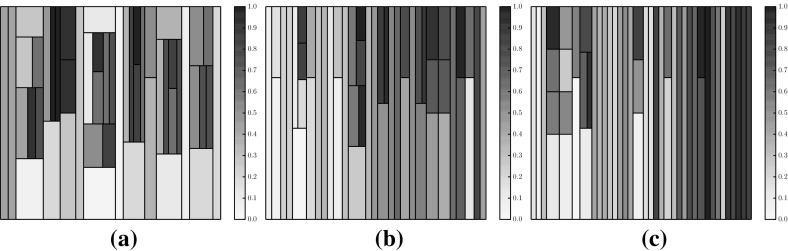
Treemaps showing populations of 50 solutions to the same 2-objective population (left), as well as populations of solutions to 3-objective (centre) and 5-objective (right) DTLZ2 problem instances. The solutions are ordered according to the first objective. **a** $M=2$, **b** $M=3$, **c** $M=5$

In order to make the treemap clearer, the order in which nodes are added to the visualisation can be controlled. Figure 5 shows three examples of ordered treemaps, in which the individuals have been ordered according to their value on the first objective. The left-hand panel shows the ordered version of the heatmap shown in Fig. 4. The other two treemaps show comparable populations for 3-objective (centre) and 5-objective (right) instances of DTLZ2. These visualisations present the individuals in a single visualisation, whereas multiple views are often required for a many-objective visualisation using a conventional approach such as a scatter plot. That said, particularly in the 5-objective case, there is an obvious lack of structure in the visualisation. Many of the individuals are mutually non-dominating and do not dominate other members of the population. This means that the majority of the population is a direct child of the root node; this is because of the aforementioned lack of discrimination provided by dominance for many-objective individuals. Given a case in which the entire population was mutually non-dominating the treemap would consist entirely of Pareto-optimal columns, and would impart very little information. We consider an approach to ameliorate this later in this paper.

### Circular treemaps

Though the procedure outlined above produced treemaps with which it was possible to view the relative quality of solutions, the arrangement of nodes made it difficult to observe the dominance relationships between dominated and dominating nodes. In this work, we propose a new treemap layout algorithm that addresses this issue. As noted in [45], there is no requirement for a treemap to follow the rectangular layout that is often used. We therefore consider a layout in which Pareto shells are arranged as layers within a circle. The outermost layer comprises the non-dominated individuals, the next layer comprises the second shell, and so on. As before, the space allocated to a node reflects that node’s importance. In the case of the non-dominated layer, this defines the proportion of the total layer that the solutions occupy. For child nodes, it defines the amount of its parent’s extent that the child occupies. By constraining child nodes to lie within their parent’s extent their dominance relationships are much clearer. As in the case of square treemaps, information is conveyed by the size of a rectangle representing a node; an individual with a large number of child nodes is represented by a larger node than one with a small number of dominating individuals, and the thickness of each layer decreases to show the diminishing importance of each subsequent Pareto shell. We note that the construction of these visualisations is similar to the *icicle plot* [29], which arranges clusters of nodes together so that they descend, in a similar way to how nodes here are arranged inwards. Both methods provide a similar view on the data; the circular design used herein is preferred as it keeps the extent of the visualisation constrained to a smaller space.

The right-hand panel of Fig. 4 illustrates the sample DTLZ2 population shown in the other two panels, as discussed earlier, with a circular treemap. The tree representation of the individuals is the same, only the arrangement of the visualisation has changed. Using this visualisation of the population it is much easier to observe the dominance relationships. For example, considering the highlighted region *A*, it is clear to see that the non-dominated individual shown in the outer ring dominates two individuals, one of which dominates another one individuals in the third Pareto shell while the second dominates two shell-3 solutions. While this information was presented in the square treemap, it is much more readily observed in the circular version. This is primarily because the Pareto shells have their own layer, and as such it is much easier to see which nodes are parents and which are the children of that node. There are examples in Fig. 5 where this information is not clear at all because there are two or more similar nodes in a region of the treemap describing a node and its children.

**Fig. 6:**
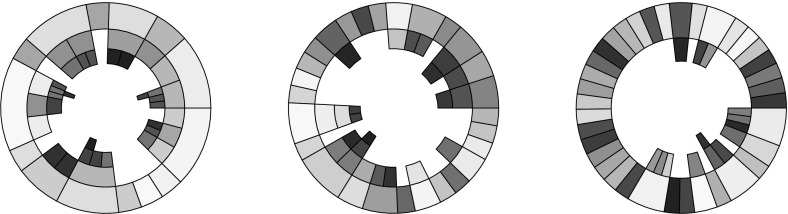
DTLZ2 sample solutions; the populations shown are for $M=2$ objectives (left panel), $M=3$ objectives (centre panel) and $M=5$ objective (right panel)

Figure 6 shows circular treemaps for the 2-, 3- and 5-objective DTLZ2 populations. Again, these visualisations clearly display the relationships between individuals and those that they dominate, however the effect of increasing numbers of objectives can be seen in the 5-objective case. The number of dominated individuals within the population is reducing, and as such the number of non-dominated individuals residing in the outer ring is increasing. As was the case with the square treemaps, this greatly reduces the usefulness of the treemap as a visualisation method. Given the prevalence of many-objective optimisation problems and the continually increasing interest in many-objective optimisation algorithms, it is important to consider methods by which treemaps can be used to visualise the data arising from such problems and algorithms.

## Many-objective mutually non-dominating sets: sunbursts

The treemaps demonstrated in the section above are suitable for representing populations of solutions in which some of the solutions are dominated, such as the search population of a MaOEA. Another common use case is to visualise the solutions resulting from the execution of such an algorithm. Generally, these solutions represent the best approximation to a given problem’s Pareto front, and as such are mutually non-dominated. Two problems occur when trying to visualise mutually non-dominating objective vectors with the scheme outlined above. First, the tree construction procedure begins by performing non-dominated sorting on the population. If all of the solutions are mutually non-dominating, then they will all belong to the first Pareto shell, and the resulting treemap will comprise a single ring. This is not very informative to a decision maker, as it does not assist them with differentiating between the solutions in their approximated Pareto front. The second issue is that some of the solutions are given substantially smaller regions within the treemap, to indicate that they are less significant and concentrate the decision maker’s attention on the solutions of higher quality. This is an advantage of the proposed treemaps when dealing with dominated solutions, as typically a decision maker will be primarily interested in those residing in the superior Pareto shells, whose individuals are represented more prominently in the treemap. In the case of a mutually non-dominated set, these “inferior” solutions do not exist. Such containment methods are not suitable in situations such as this where the deeper nodes are important to the visualisation (as opposed to the case described earlier, in which the outer rings were the most important).

### Producing sunbursts from quad trees

The first of these issues, representing a mutually non-dominating population with a tree, has been tackled within the evolutionary optimisation literature—though not from the standpoint of visualisation. A potentially computationally expensive task within a MaOEA is identifying whether a newly evolved solution is dominated by, or dominates members of the current Pareto front approximation. A naive approach is to compare each solution of the archive and check the dominance relationships between them and the new solution. Various attempts have been made to leverage the lower complexity of lookup within a tree. Two examples of non-dominated trees are [19, 33].

The algorithm used in this work constructs a quad tree from a many-objective mutually non-dominating tree and was proposed in [41]. The process relies on the notion that one individual is the *successor* to another—which aligns well with the requirement for each node to have one parent, as already been discussed herein. One solution $\mathbf {y}_i$ is called the ${\varPsi }$-successor of $\mathbf {y}_j$, where ${\varPsi }$ is computed as follows:

5 $$\begin{aligned} {\varPsi }_m = \left\{ \begin{array}{ll} 1\qquad \text{ if } \ y_{im} \le y_{jm}\\ 0\qquad \text{ otherwise }. \end{array} \right. \end{aligned}$$

Having computed a ${\varPsi }_m$ value for each objective, the overall value ${\varPsi }$ is computed with:

6 $$\begin{aligned} {\varPsi }= \sum ^M_{m=1}({\varPsi }_m)2^{M-m} \end{aligned}$$

The tree begins with the first member of the population being added as the root node. Then, as each new solution is added, a check is made to see if there is already a ${\varPsi }$-successor in the tree. If there is not, then the solution is added to the tree as a child of the root. Otherwise, the same check between the new solution and the root’s existing ${\varPsi }$-successor to see if the new solution can be that node’s child, and so on until a node is found for which there is no ${\varPsi }$-successor and the solution is added to the tree.

The second issue, relating to the difficulty in seeing the inner-most nodes of a circular treemap, is addressed by using a slightly different visualisation. The *sunburst* [40] operates in the same way as a circular treemap, representing a node’s children within the extent of the parent, however it places the root node at the centre and child nodes emanate from it. This allows all of the nodes to be visible, and as such no part of the the tree is implicitly less important than any other. The size of the node is again determined by the number of child nodes beneath it, as was done in the treemaps earlier. This work introduces the use of sunbursts for visualising many-objective populations.

### Sunburst examples

Several demonstrations of sunburst plots representing mutually non-dominating sets follow. The examples are drawn from a number of sources, including 3- and 10-objective *benchmark approximation sets* [43], samples from the discontinuous Pareto front of a test problem from the literature [14] and the solution set generated by using a MaOEA to optimise a real-world benchmark problem comprising 9 objectives, identifying good designs of radar waveforms [24].

#### 3-objective BAS & DTLZ6 plots

**Fig. 7:**
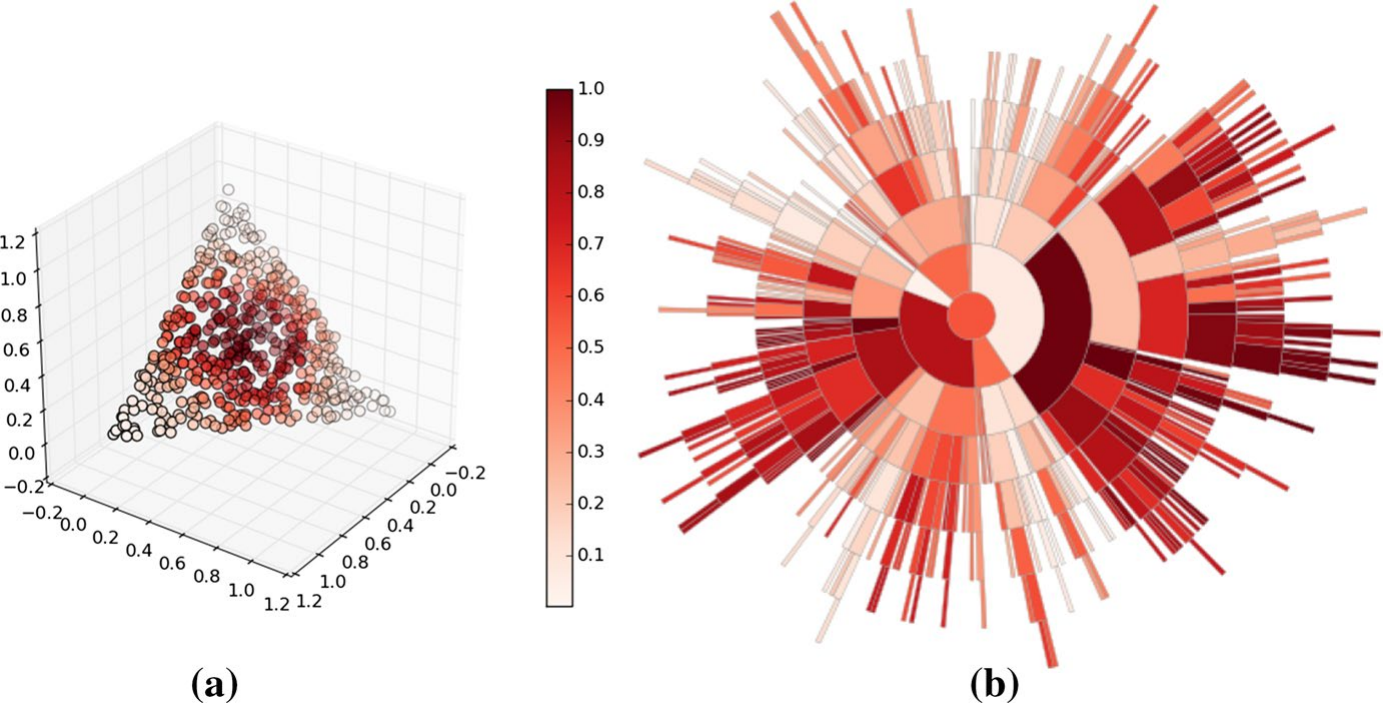
The objective vectors of a 3-objective linear BAS (left) and the corresponding sunburst plot (right). Colour indicates average rank, normalised to (0,1). **a** Objective vectors, **b** sunburst (Color figure online)

As with the treemap visualisations of dominated populations, the mutually non-dominating sets to be visualised with sunburst plots are demonstrated using solutions to a known test problem (DTLZ6 [14]). In addition, two benchmark approximation sets (BASs) are used. BASs were introduced by [43] in order to facilitate more systematic analysis of visualisation methods. They propose two BASs, one of which is linear and is used in this work. It provides known distributions of points across a mutually non-dominating set and its solutions within are distributed uniformly. The left-hand panel in Fig. 7 shows the distribution of the two BASs, each of which comprises 500 points. The points are coloured according to their average rank, with white indicating a good rank and dark red a poor rank. Highly ranked points are clustered in the corners. The right-hand visualisation shows the quad tree representation of the linear BAS. As can be seen, there is a strong degree of correlation among the rank of the nodes, with regions of very poor solutions and regions of stronger solutions. Displaying the population in this way is a useful way of guiding the decision maker towards those more highly ranked regions of an estimated Pareto front without providing them with a specific solution to select, encouraging them to explore interesting regions of the solution set.

**Fig. 8:**
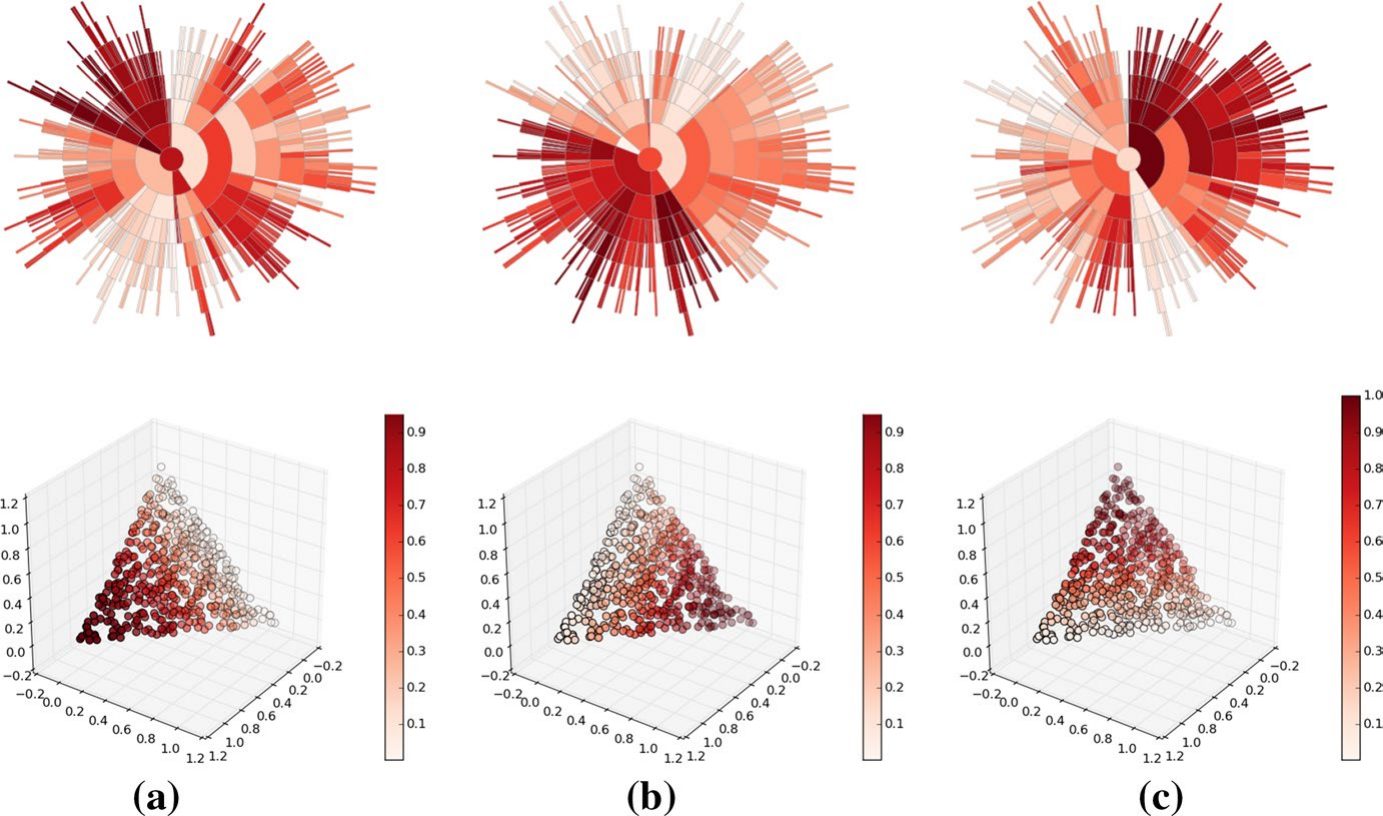
Sunburst plots of the 3-objective linear BAS along with the population. Each pair of plots is coloured according to a different objective (1, 2 and 3) to show the trade-off between the objectives and demonstrate how this is viewed in the sunburst. **a** Objective 1, **b** objective 2, **c** objective 3 (Color figure online)

An important aspect of visualisation is interaction, and one of the strengths of this approach is that it lends itself to updating to show different properties of the population. Assuming a preference on the part of the decision maker for one or more objective, having undertaken the computational expense of arranging the sunburst the nodes can be quickly recoloured. Figure 8 shows three more versions of the same sunburst plot, with each showing the solutions’ rank according to one of the objectives (the left-hand plot shows the solutions ranked according to their value on the first objective; the second shows the rank according to objective two, and the right-hand plot shows objective three). In each case, a different region of the sunburst is shown to have the highest rank for a given objective, demonstrating the trade-off between the objectives.

**Fig. 9:**
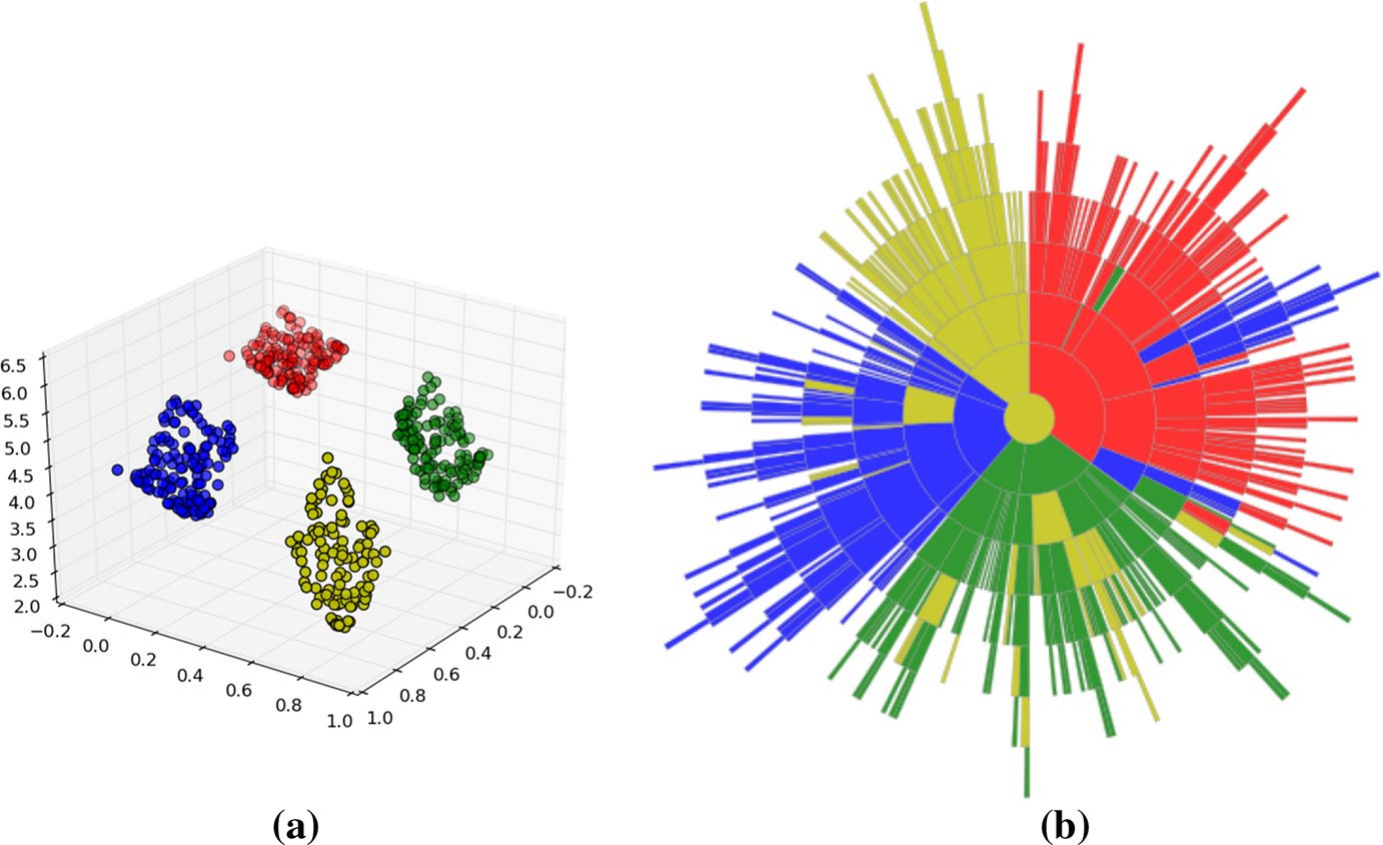
A sunburst showing 500 solutions drawn from the Pareto front of DTLZ6. Each disconnected region is coloured differently, and regions are clearly visible within the sunburst. **a** Objective vectors, **b** sunburst (Color figure online)

The final 3-objective example is drawn from the DTLZ6 test problem. This problem features a discontinuous Pareto front, with the number of discontinuous regions depending on the number of objectives forming an instance of the problem. In the 3-objective case the problem has four disconnected regions, shown in the left-hand plot of Fig. 9; each region is shown in a different colour. The disconnected regions are clearly visible in the sunburst plot, with a small degree of overlap (a small number of yellow and blue nodes appear amongst the nodes belonging to other disconnected regions). This further demonstrates the ability to identify regions of the Pareto front, beyond that demonstrated in the BAS examples above. As well as demonstrating that the tree construction proposed herein preserves the spatial characteristics of the Pareto front, the information provided to a user by colouring according to a cluster or disconnected region is of use to a decision maker. While there has been no effort to weight objectives in this work, the decision maker will have preferences that guide their selection of a solution. By categorising objectives in this way, they are guided towards regions of interest and can further explore solutions in those regions more thoroughly while ignoring solutions in areas of the Pareto front that are not of interest.

**Fig. 10:**
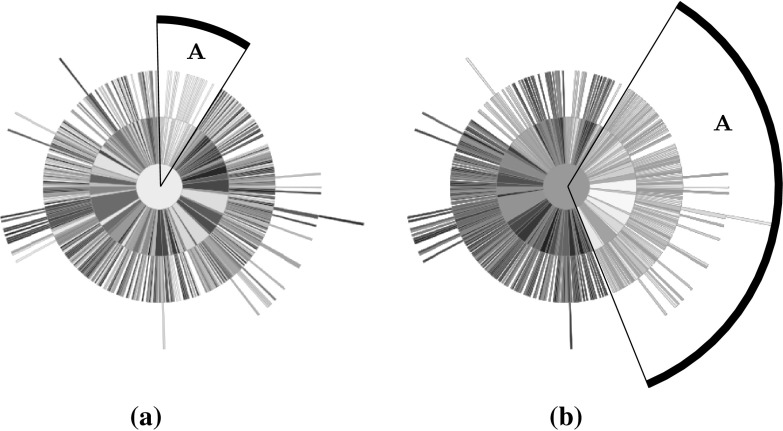
Two views of the same 10-objective linear BAS. In the left-hand example, node’s are coloured according to their corresponding solutions normalised score on the first objective. The right-hand example shows the same information, with nodes coloured according to objective 4. In both cases, solutions minimising the respective objectives, shown by lighter nodes, are clustered within the region *A*. **a** Objective 1, **b** objective 3 (Color figure online)

#### 10-objective BAS plots

Having demonstrated the potential for using the sunburst visualisation of a quad tree to identify regions of interest within a mutually non-dominated set, it is important to consider how well the method scales to larger number of objectives. The methods used to generate BASs are scalable to any number of objectives, and a 10-objective linear BAS is constructed. The set consists of 500 solutions. Two instances of a sunburst representing the BAS are shown in Fig. 10. In each case, the solutions are coloured according to their score on two of the objectives (objective 1, left, and objective 3, right). In both cases, regions (marked *A*) have been highlighted that indicate a cluster of solutions with a lower score on that objective than is found elsewhere in that sunburst. Though the clustering is less clear than it was in the case of the 3-objective populations, especially in the right-hand case, it is important to note that these populations are more complex given the higher number of objectives. Despite this it is possible to observe this relationship in the high-dimensional space, which indicates that the sunburst is a useful many-objective visualisation tool. As noted above, it is a trivial matter to recolour the nodes of the sunburst, and thus the visualisation could easily be incorporated into an interactive tool where it would be used to explore a high dimensional population such as this.

#### 9-objective radar plots

**Fig. 11:**
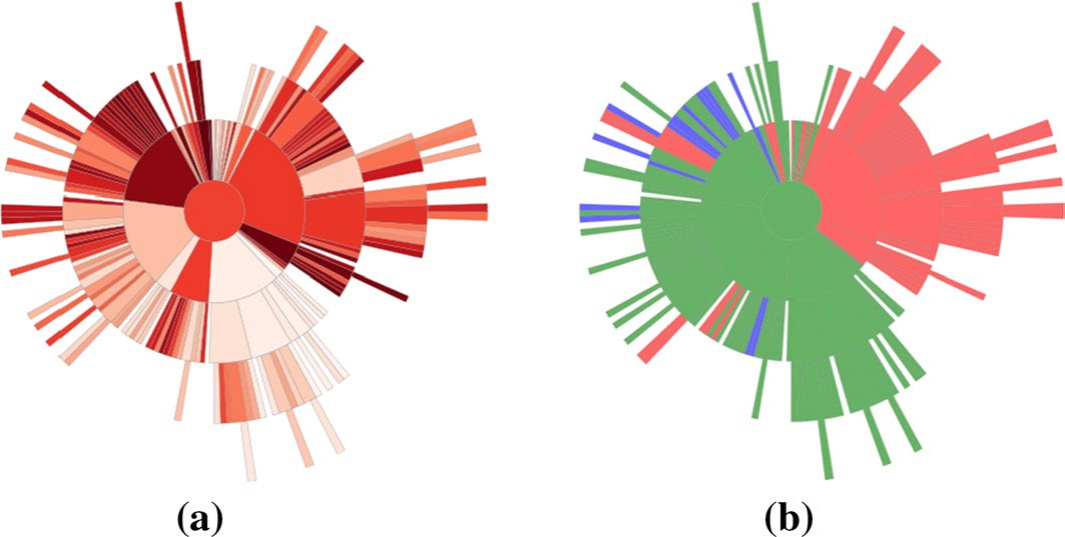
Two sunbursts visualising the radar archive. The left-hand plot colours solutions according to average rank, while the right-hand plot shows the objective class on which each individual has the best rank. Red indicates a range objective; green indicates a velocity objective, while blue represents the transmission time. The vast majority of the range objectives are contained within their own cluster, while the velocity and transmission time are intermingled—they are known to be well correlated. **a** Ranks, **b** objective class (Color figure online)

The final demonstration of mutually non-dominating treemaps is for a real world test case. Proposed by Hughes [24], this population of mutually non-dominating solutions optimises a 9-objective radar waveform design problem. A solution to the problem comprises 12 *pulse repetition intervals*, and the problem is described by nine objectives; four optimise the range at which objects can be detected, another four optimise the velocity at which objects can be detected, and the final objective minimises the transmission time of the complete waveform. Details of the optimisation procedure can be found in [24]. Figure 11 presents two versions of a sunburst. In the first, the solutions are coloured according to average rank. As has been the case in earlier examples, those solutions with the best rank are clustered together—the lower-right segment of the plot comprises predominantly light coloured nodes. The second colours the solution according to the type of objective it best optimises. The solutions were converted to rank coordinates, placing each objective on the scale $1,\ldots ,N$, and the best rank for each solution was identified. This information was used to colour the solutions according to the class of objective they best optimise, so that those best optimising range objectives are coloured red, velocity objectives are green, and the transmission time objective is shown in blue. The visualisation illustrates a known correlation between the objectives. The range objectives are anti-correlated with the velocity objectives, and in the sunburst plot they are placed away from each other, with the exception of a few range nodes within the velocity region. The transmission time objective is correlated with the velocity objectives, and the blue nodes are within the green velocity region. With this information, the trade-off between objectives can be seen. It is not possible to have a solution that simultaneously optimises range, velocity and transmission time objectives. Those optimising range objectives well are gathered away from those optimising the velocity and transmission time objectives, and offer poor performance on those other objectives. As was the case with the DTLZ6 example, this information will be used in combination with the user’s preferences, and will better inform their identification of regions of interest within the Pareto front.

**Fig. 12:**
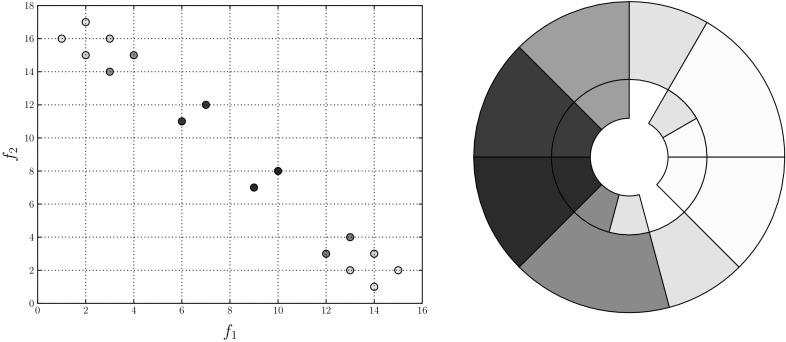
Visualising population diversity using a treemap. Those individuals in the centre of the population with a larger crowding distance are shown in the centre of the treemap. Individuals are ordered according to their objective value for the first objective (Color figure online)

**Fig. 13:**
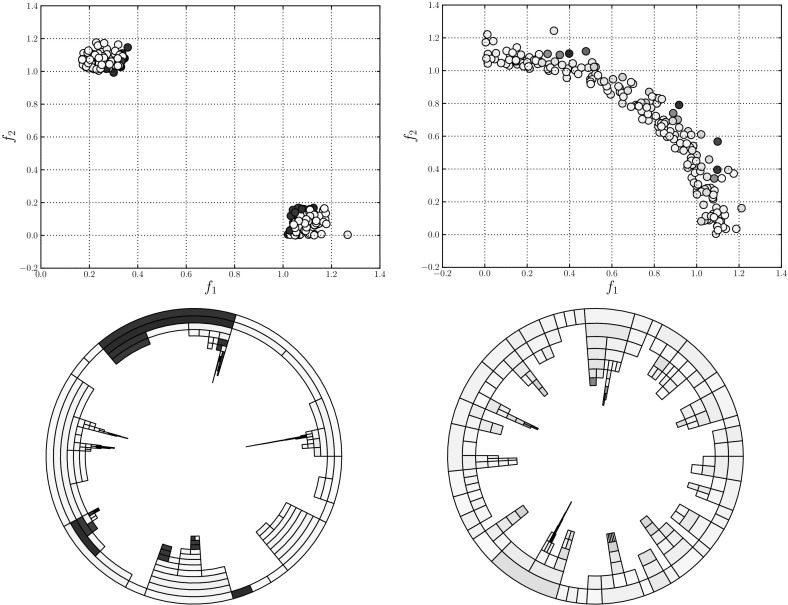
Two circular treemaps showing coverage of the Pareto front according to crowding distance for two sets of samples drawn from an instance of DTLZ2. The top treemap shows the discontinuity in the solutions by the dark solutions. The lower population contains no such discontinuity, and solutions are more evenly coloured (Color figure online)

### Visualising diversity and convergence

Beyond the visualisation of solution quality, aspects of evolutionary optimisation such as population diversity and convergence are an important consideration. We consider an approach in which solution diversity is evaluated in objective space using the crowding distance measure employed within the selection operator of NSGA-II [13]. Crowding distance identifies the distance between a solution and its next nearest neighbour on each objective. In Ref. [47] demonstrated the use of evaluating Euclidean distances between parameter values to consider diversity in solution space. We note that this is a sensible alternative to the approach taken herein, and that a range of measures might be appropriate in order to consider different solution representations (e.g., permutation-based approaches).

Figure 12 illustrates this approach on a sample population comprising two Pareto shells arranged on the plane. In both shells, the distribution of points is uneven, so that the points in the centre of the shells are spread out and those at the edges are closer together. The effect of this is to give those central individuals a larger crowding distance than those at the edge, and that can be clearly seen in the treemap shown in the lower panel of Fig. 12. The individuals in the centre of the shells are those on the left-hand side of the treemap, coloured dark blue in both shells. The edges of the population are located on the right-hand side of the visualisation. A second sample population is shown in Fig. 13. This data was generated by sampling from the true Pareto front of DTLZ2. In a *P*-dimensional chromosome, the first $M-1$ parameters control the position of a solution on the true Pareto front and the rest control the distance of the solution from the front. The sampled solutions were moved away from the Pareto front by adding a small amount of random noise to these distance parameters, to create a population with dominated solutions. Two variants of this population are shown. In the first, shown on the left-hand side of Fig. 13’s top panel, a large discontinuity has been induced by placing the individuals at the extreme edges of the population, essentially forming two clusters of solutions. The individuals on the inner edges of these clusters have a large crowding distance, and these individuals are clearly visible in the corresponding treemap, shown in the centre panel; they are the dark blue individuals. Conversely, the second population contains no such discontinuity, and the solutions are all much closer together. That population’s treemap, shown in the bottom panel of Fig. 13, has a much more uniform distribution of colours, and the colours are much lighter.

In addition to visualising diversity, it is also important to present an idea of the extent to which the population has converged. Here we use the age of the archive to demonstrate the extent to which the algorithm has converged, based on the idea that once an algorithm has converged it will contain solutions that are no longer replaced, and the presence of such “older” solutions can be highlighted in the treemap. In order to demonstrate that a treemap can be used to show the convergence of a population we optimise a 3-objective instance of DTLZ2 with a series of MaOEAs designed to show different convergence characteristics. In each case, the underlying algorithm is a simple ($\mu +\lambda$)—evolution strategy (ES). The exact operation of the base algorithm is outlined in Algorithm 1. The algorithm initialises a random population of *N* solutions, which are evaluated and used to initialise an elite archive to represent the current approximation of the Pareto front. At each generation, the population is copied and each individual is mutated with an additive Gaussian mutation drawn from $\mathcal {N}(0,0.1)$. The solutions are evaluated and the archive updated (any that the new child dominates are removed, and if the child is not dominated by the archive then it is added) before elitist selection is performed on the combined parent and child populations. The first ES employs uniform random selection, designed to show very slow convergence with reasonable diversity. The second employs Pareto sorting, which is designed to offer better convergence and maintain the diversity of the population, while the final optimiser uses average rank selection, retaining the top *N* solutions in combined parent and child populations. This is a selection strategy known to promote premature convergence to a small region of the Pareto front [21]. 
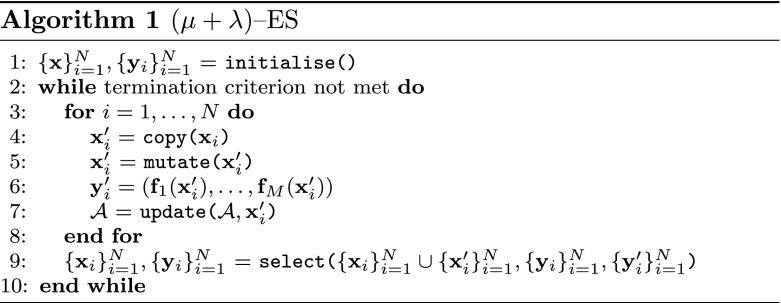

**Fig. 14:**
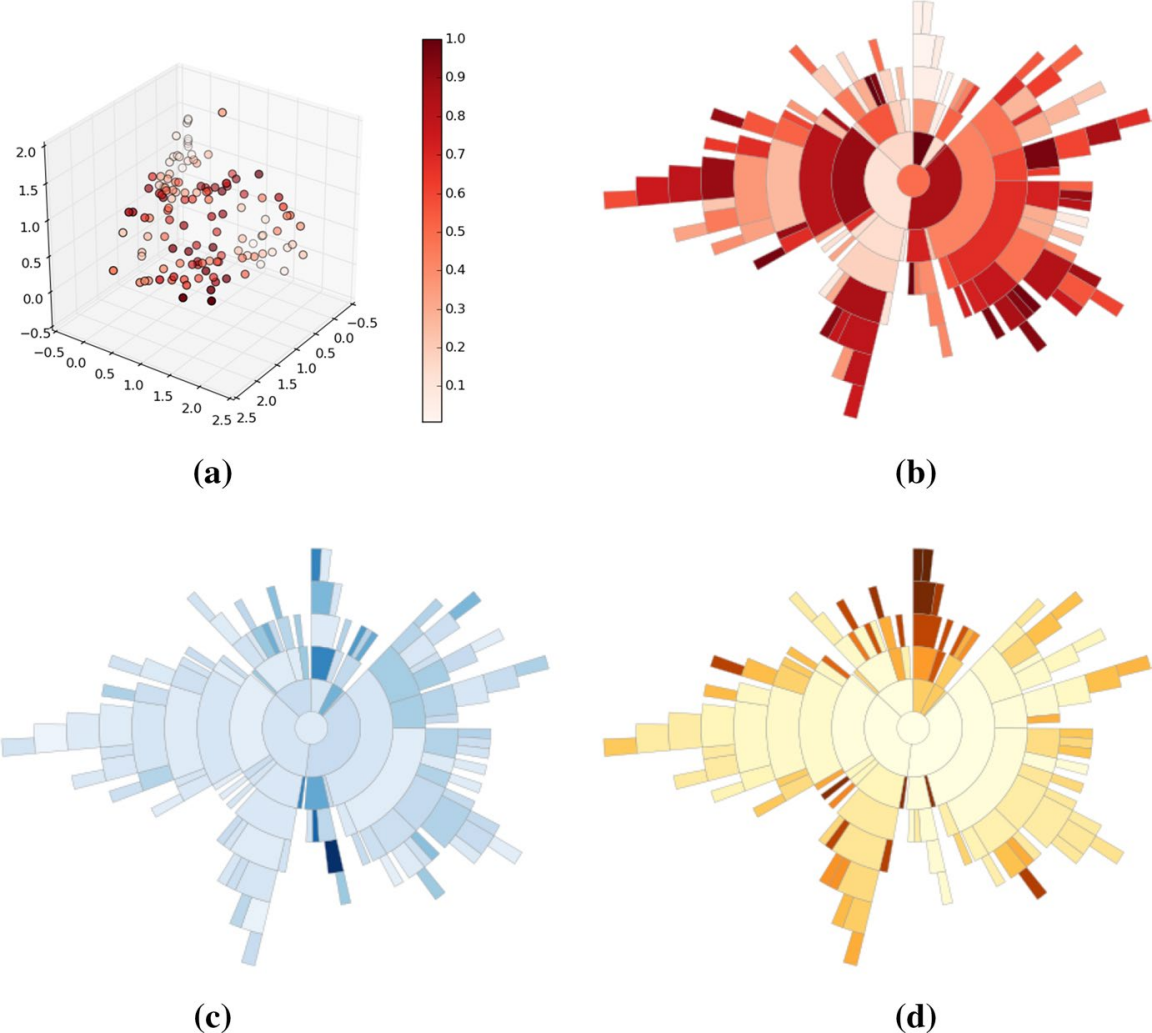
Optimisation result using random selection. **a** Solutions, **b** rank, **c** diversity, **d** convergence (Color figure online)

**Fig. 15:**
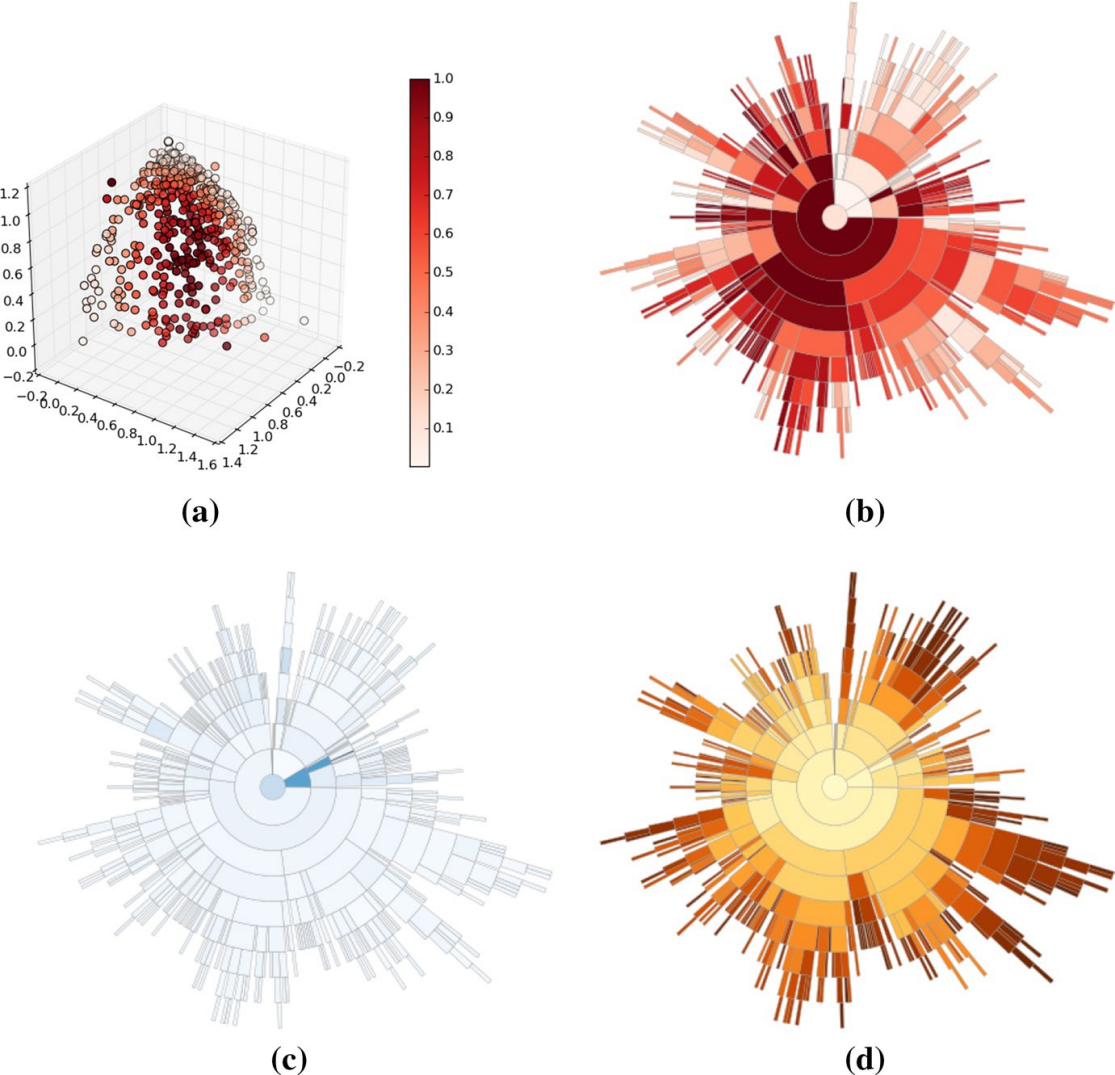
Optimisation results using Pareto sorting selection. **a** Solutions, **b** rank, **c** diversity, **d** convergence (Color figure online)

**Fig. 16:**
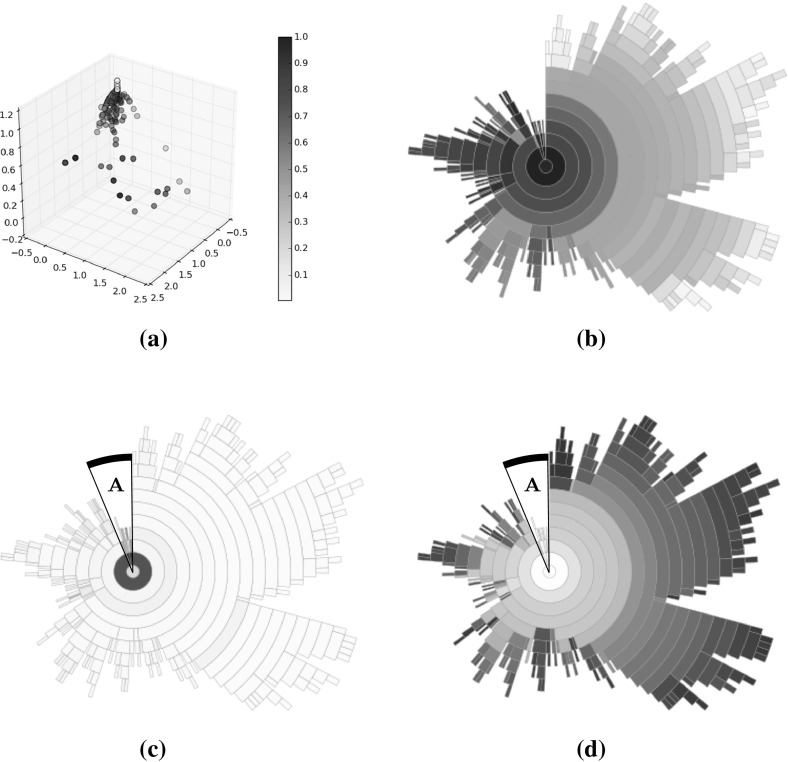
Optimisation results using average rank selection. The extreme convergence of some solutions has caused a deeper tree than was seen for either the random selection or Pareto sorting examples. Region *A* within the sunburst indicates a group of poorly converged solutions from early on in the optimisation. **a** Solutions, **b** rank, **c** diversity, **d** convergence (Color figure online)

Figures 14, 15, 16 illustrate sunbursts of the results of these optimisation experiments. Each figure refers to a different selection mechanisim—Fig. 14 shows the random selection case; Fig. 15 shows the Pareto sorting example, and Fig. 16 shows the average rank results. The top-left panel shows the objective space image of the objective vectors. The solutions in all three final archives were ordered using average rank, and this information was used to colour the individuals in the population view as well as the sunburst visualisation next to it. The bottom row shows the same sunburst representation of the archive; the left-hand plot is coloured according to crowding distance to show diversity, while the right-hand shows the age of the solution to indicate convergence. In the case of the diversity, the best results were achieved by the Pareto sorting algorithm. That algorithm’s sunburst has the lightest colouring, indicating that the crowding distance values are much more uniform and the solutions are better spread across the Pareto front. In both of the other cases, the treemaps feature dark blue colouring more, meaning that those Pareto front estimations contain more distant solutions. This is particularly the case for the average rank optimiser, which has explored very little of the Pareto front. This is supported by the convergence sunbursts, which, again, display the (normalised) age of the solutions in the archive. The age is specified by the generation number in which the solution was archived, so a low number indicates an old solution that was generated early on in the optimisation process. The premature convergence exhibited by this algorithm is shown clearly by the large group of solutions, shown in dark brown at the top of the sunburst, which have been in the archive since the beginning of the optimisation procedure. These correspond to the random solutions scattered at the bottom of the top panel’s scatter plot and are shown in region *A*. They correspond to the solutions with the largest crowding distance in the diversity sunburst. Beyond examining the colouring of the visualisations, these sunbursts convey more information through their structure than has been seen before. From examining all three it can be seen that the number of layers provide an indication of the degree to which the population has converged—the random selection algorithm has 10 layers, compared to 12 in the Pareto sorting example and 20 in the prematurely-converged average rank case. There are also fewer missing regions in the sunburst representing the “ideal” case, using Pareto sorting, with the visualisation tending more towards the full circle shape seen in earlier examples sampled from the Pareto front and in the BAS examples.

## Validation

As well as discussing the topological features that the treemaps and sunbursts allow the user to observe, it is important to quantify how useful the proposed methods are. To do this, a small user experiment was conducted to assess the extent to which the user can identify solutions of interest, as well as examining the accuracy of their selection. Using the nomenclature from [30], a *controlled experiment* was carried out. Sunburst plots were pit against three other many-objective visualisation methods drawn from the literature. These are seriated heatmaps [46], parallel coordinate plots [25] and multidimensional scaling (MDS) [37] constructed using the dominance distance [46]. These methods were chosen as a cross-section of existing methods from the evolutionary computation visualisation literature, and include a method that shows the actual objective values (parallel coordinate plots); a method based on all *M* objectives (heatmaps); and a dimension reduction method (MDS). Three sets of 50 mutually non-dominating solutions were constructed from datasets seen previously herein—the linear BAS, spherical BAS and samples drawn uniformly at random from the radar waveform optimisation solutions (giving different types of Pareto front geometry and different numbers of objectives (10, 3 and 9 objectives, respectively)). The 50 samples were generated at the start of the experiment, meaning that each user saw different datasets. That set of 50 samples was then displayed with each of the four visualisation types.

Nine users were shown each dataset using all four visualisations. The users were Computer Science researchers, and all had prior knowledge about mutually non-dominating sets. No other prior selection of user was performed, and participants were from a range of ages, and were both male and female. The experiment was conducted in a lab, and users were presented with visualisations on a screen. Their task was to identify the best solution, according to the average rank measure, in each case. The visualisations were benchmarked in two ways: the amount of time taken for the user to make their selection (recorded automatically by the visualisation software), and the distance in rank space between their selected solution and the best solution they could have chosen. Assuming the user selects the solution $\mathbf {y}_i$, this distance is calculated as follows. First, the solution is converted to rank-coordinates to produce a vector of ranks $\mathbf {r}_i$:

7 $$\begin{aligned} \mathbf {r}_i = (r_{i1}, \ldots , r_{iM}), \end{aligned}$$

where $r_{im}$ is again the rank of the *i*-th solution on the *m*-th objective. Then, assuming the best solution (identified with the average rank procedure, outlined earlier) is represented by the rank vector $\hat{\mathbf {r}}$, an error term is computed with:

8 $$\begin{aligned} E = \sum ^M_{m=1} |r_{im} - \hat{r}_{im}|. \end{aligned}$$

In the ideal scenario, the user selects the best solution and *E* is 0. The further away from the ideal the user is, the worse their score will be.

**Fig. 17:**
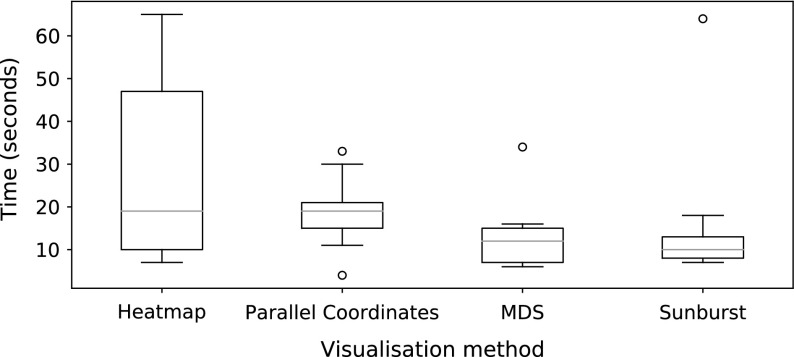
User response times for the four visualisations compared

**Fig. 18:**
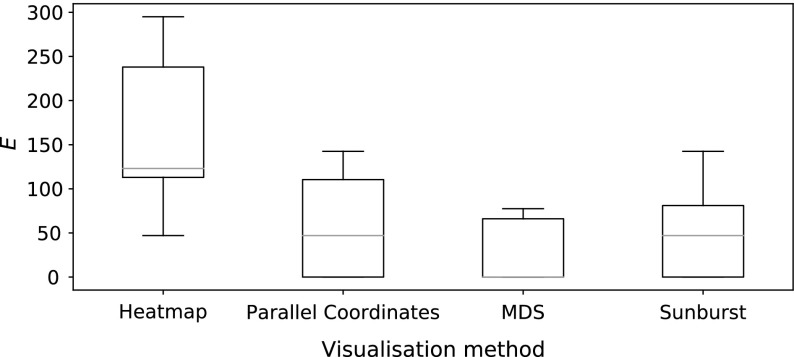
Accuracy scores for the four visualisations compared

Figure 17 illustrates the timing results for each user. As can be seen, the maximum response time was just over one minute, with most decisions being made within twenty seconds. It took users considerably longer to identify the best solution, and users were slightly faster with the sunburst than the MDS. Anecdotally, one user commented that the extra information provided by the size of the node in the sunburst made it easier to use than the MDS projection, in which each solution was represented with a circle of equal size. Further anecdotal evidence from users suggested that they found the additional information available from viewing the exact objective values in the parallel coordinate plots and heatmaps confusing, which is supported from the time taken shown here. It is important to note that the visualisations were presented in the same order, and therefore some learning may have taken place on the part of the user that enabled them to use later visualisations more effectively. The accuracy results shown in Fig. 18 show a similar trend. Again, the heatmaps are the hardest for users to identify the best solution with, but here parallel coordinates plots are shown to be less distinguishable from MDS and sunbursts than they were considering time taken. MDS is the most accurate way of identifying the most highly ranked solution. Though this result shows that more accurate results can be obtained with MDS than with the sunburst visualisations, we note that MDS requires the potentially expensive step of computing pairwise distances between the solutions before the 2-dimensional projection can be constructed. Were a MaOEA using a tree-based archive to store its approximation of the Pareto front to be used to generate the solution set, the visualisation task with in the sunburst case would simply be partitioning the space and colouring the nodes.

## Discussion

The sections above demonstrate that both treemaps and sunburst plots can be used to convey useful information within evolutionary computation. In order to contextualise the methods with others used in the field, they are evaluated using the framework proposed by Tusar and Filipić [43]. They characterise nine properties of a population visualisation: the preservation of (1) dominance relations, (2) front shape, (3) objective range, and (4) the distribution of vectors; (5) robustness; (6) the ability to handle large sets; (7) the simultaneous visualisation of two or more populations; (8) scalability; and (9) simplicity. The visualisations proposed in this work are formed of two components—the treemap or sunburst visualisation itself and the underlying tree structure. The proposed methods are discussed with respect to the approaches taken herein, and may not apply to other tree structures.

The treemap visualisations preserve some dominance relations by including the dominated child within the extent of its parent solution. Those that are discarded in the tree construction process are lost, however care is taken to preserve the relation between the child and the dominating solution with which it is closest using the dominance distance. The sunburst visualisations were used to show mutually non-dominating sets, so in terms of dominance all of the solutions are incomparable. Both visualisations are capable of preserving dominance relations, depending on the type of tree structure used to store the individuals. It is also possible to illustrate distribution of objective ranges can be included with colour. In this work the objective ranges have been normalised, however as has been discussed it is computationally cheap to update the colour of nodes. The distribution of vectors is shown both through the arrangement of nodes and by applying a colour indicating distances between points. In the mutually non-dominating sets generated by the three MaOEAs different distributions of solutions were obtained; the well converged diverse population had a rounded arrangement, while the other two (showing poor convergence and premature convergence) had gaps. Both visualisations are capable of supporting large populations, both in terms of the number of solutions and the number of objectives; hence, they meet both the criteria relating to handling of large sets and scalability. In terms of simplicity, the treemaps and sunbursts are both constructed with a recursive function that is called linearly with the number of individuals in the population. The complexity of tree construction is also a consideration; the original purpose of storing mutually non-dominating sets in tree structures was to enable fast lookup for checking dominance relations, which means the computationally expensive operations are carried out during tree construction. That said, the algorithm used herein completes tree construction in polynomial time. Thus, both treemaps and sunbursts meet the simplicity criterion, with the proviso that additional complexity can be introduced depending on the desired colour scheme.

Three of the characteristics are not observed in treemaps or sunbursts. The shape of the Pareto front is not conveyed in either the treemap or sunburst visualisations. According to [43], robustness refers to the ability to add solutions without changing the existing population. The visualisations herein are not robust because an additional solution will change the structure of the population. Finally, both treemaps and sunbursts represent a single population, therefore they are not suitable for comparing between different solution sets.

Beyond contextualising the methods in terms of the properties of a population visualisation, it is important to compare them to other methods in terms of the tasks they will be used to perform. A taxonomy of visualisation tasks was proposed [1] and we evaluate treemaps and sunbursts according to those tasks and compare them to other visualisation methods. The taxonomy provides ten tasks, some of which are not relevant to the general goal of identifying good solutions using the visualisation, but most of which are. The tasks defined by the taxonomy are: (1) retrieve value; (2) filter; (3) compute derived value; (4) find extreme values; (5) sort; (6) determine range; (7) characterise distribution; (8) find anomalies; (9) cluster; and (10) correlate.

Probably the most relevant to the overall goal are *identifying extreme values* and *sorting*. In both cases, both treemaps and sunbursts facilitate this through the colouring of nodes. In the examples demonstrated, we have shown that the nodes can easily be coloured according to the overall quality of a solution, or by the solutions’ value on an individual objective. In each case, the decision maker can look for the extreme colour to identify the best solution (as well as the worst), and can use the colour gradient between the maximum and minimum to infer an ordering of solutions. In a similar fashion the *range* of each objective can be identified by colouring the solutions by the relevant objectives. We note that in this paper the visualisations have shown normalised objective values—to identify the range of the objectives it would be necessary to visualise the objective range. A similar task is evaluating the *distribution* of objective values. This can be done, again, by considering the solutions’ colour. The authors’ description of distribution analysis [1] discusses the comparison of different classes—this relates to the comparison of different objectives, which in turn leads to trade-off analysis. While it is possible to observe the trade-off between different objectives, it relies on changing the node colouring between the objectives being compared, and in the methods’ current form it is not possible to visualise this without interaction. We do not feel that this is too much of a deficiency, however it is worth considering in future work. The ability to identify *clusters* within the data has been demonstrated within the examples shown earlier. In those examples, the data were coloured using *a priori* information—which region of the Pareto front the solutions belong to in Fig. 9 and which class of objective the solutions achieved the best rank on in Fig. 11. Clusters were also visible in the visualisations coloured according to average rank, in which regions of good and poor solutions could be observed, and in those coloured according to a specific objective. Again, regions of good performance on the objective at hand were easily discerned.

Of the remaining five tasks that are not immediately facilitated by the treemaps or sunbursts, value retrieval, filtering, anomaly detection and correlation observation might easily be facilitated through interaction. The individual objective values are not present in the basic visualisation (though the visualisation is based on them), however they could be easily displayed for a selected solution alongside the visualisation. Likewise, additional work would be required to implement filtering of objective values, but this would be possible through the addition of a user interface. This feature could be extended to observe correlations by allowing composite filtering, however users are likely to be better served by providing a heatmap or parallel coordinate plot in such tasks. The same is true of anomaly detection. The computation of derived values is not something that a user engaged in tasks defined would use such a visualisation for, though, again, in theory it could be incorporated within the user interface.

## Conclusion

Hierarchical information is common within evolutionary computation. This paper has presented treemaps and sunbursts for visualising data in evolutionary computation, focussing on populations of solutions to many-objective problems. The visualisation of such data is an important task, as decision makers find comprehending solutions described by a large number of objectives difficult. Treemaps are a good choice of visualisation tool because of their flexibility. They have various degrees of freedom that can be exploited to convey the structure of a population. Though the standard form of a treemap is a square grid in which nodes are represented by rectangles, we have presented an alternative layout algorithm that is better suited to displaying the dominance relationships that characterise a many-objective population. By using circular treemaps, the parent–child (and therefore dominance) relationships are much easier to observe. In addition to showing individual quality, we demonstrated that treemaps can be used to convey other information relevant to the operation of an MaOEA, such as the diversity within the search population and how well converged the solutions are.

We have presented a novel algorithm for building a tree of multi-objective solutions so that a treemap can be rendered. Based on dominance, the algorithm is shown to be suitable for multi-objective populations, but does not scale well to deal with many-objective populations. This is because many-objective individuals are generally incomparable under dominance, so instead a tree construction algorithm from the literature was employed. Whereas circular treemaps give most prominence to those individuals in a population that are non-dominated, with less significance given to dominated solutions, a mutually non-dominating set does not have such preferential individuals. As such, a sunburst visualisation is used instead of a treemap, which sees nodes radiating out from the centre rather than inwards as is the case with circular treemaps. Both of the methods result in graphs that convey useful information. We acknowledge that, unlike some of the other methods for constructing multi-objective trees in the literature, the purpose of these trees is to be used as a basis for visualisation. Were they to be used within an optimisation process, the computational complexity would likely be an issue. That said, for the construction of a one-off visualisation this is not an issue, and we believe that they are fast enough to be used within an interactive visualisation too.

The main advantage offered by the approaches described herein are their flexibility. We have used three visualisation methods in combination with two tree representations of data arising within evolutionary computation to visualise aspects of that data, however the use of these methods is far from restricted to the approaches we have taken. As discussed in Sect. 2, other approaches to representing data as trees have been taken in evolutionary computation, and any of these tree representations could form the basis of a treemap visualisation. Likewise, the visualisation literature contains a plethora of approaches to arranging treemaps. Though the circular treemap proposed here is designed specifically for use in evolutionary computation, to illustrate dominance relationships between multi- and many-objective individuals, the selection of layout algorithm is largely problem specific. An aspect of future work is to consider other areas of evolutionary computation in which treemaps might be productively used, and design new ways of illustrating this hierarchical information.

In addition to considering other applications of treemaps and sunbursts, several aspects of future work are worthy of consideration. In terms of the mechanics of the visualisation, these fall into two groups. In the first, the tree used as the basis of the visualisation would be enhanced. As has been discussed, one of the benefits of the proposed method is that the visualisation is completely decoupled from the underlying tree, so it would be useful to consider whether there are characteristics of mutually non-dominating sets that can be more effectively represented by using a strategy other than the successor-based quad tree demonstrated herein. Beyond this, there may be other data structures in use within MaOEAs that might inform or inspire a visualisation in the same way that this work was inspired by research into the use of trees to represent populations with a treemap. The second area of future work would consider alternative layout algorithms. This work has shown useful visualisations of solution sets, however some of the characteristics of the sets were lost, such as their *shape*. Mutually non-dominating sets are characterised by their shape; this can be, for example, linear, convex or non-convex, and is a piece of information that this work does not consider. Though it was possible to observe the trade-off between objectives by updating node colourings to show different objective values, an ideal visualisation would incorporate this within a single visualisation without needing multiple views. We feel that the potential of presenting evolutionary computation data in this way is an exciting prospect, and likely to be extremely useful to evolutionary computation practitioners.

A final extension that we are currently exploring is how the visualisation can be more thoroughly evaluated. Elsewhere in evolutionary computation, such as algorithm development, rigorous benchmarking of methods is employed. In the realm of visualisation this has not historically been the case, and we are currently investigating how evaluation methods within the visualisation field might be applied within evolutionary computation to facilitate a more scientific investigation of methods such as those proposed herein, beyond examples such as the small user experiment used to gather quantifiable data used in this work. The study included herein would benefit from a random ordering of the visualisations, in order to eliminate the potential for visualisations presented later in the study to benefit from greater understanding on the part of the user. We feel that such investigation will lead to the development of much better visualisations for the wider evolutionary computing field.
